# 1-(2-Hydr­oxy-4,5-dimeth­oxyphen­yl)propan-1-one

**DOI:** 10.1107/S1600536809041798

**Published:** 2009-10-23

**Authors:** Changchun Cen, Guangying Chen, Changri Han, Xiaoping Song

**Affiliations:** a Hainan Provincial Key Laboratory of Tropical Pharmaceutical Herb Chemistry, College of Chemistry & Chemical Engineering, Hainan Normal University, Haikou 571158, People’s Republic of China

## Abstract

In the title compound, C_11_H_14_O_4_, isolated from the stems of *Trigonostemon xyphophylloides*, an intra­molecular O—H⋯O hydrogen bond helps to establish an essentially planar conformation for the mol­ecule (r.m.s. deviation = 0.044 Å).

## Related literature

For botanical and biochemical background, see: Tempeam *et al.* (2005 ▶); Chen *et al.* (2009 ▶). For medicinal applications of this family of compounds, see: Chuakul *et al.* (1997 ▶); Tempeam *et al.* (2002 ▶).
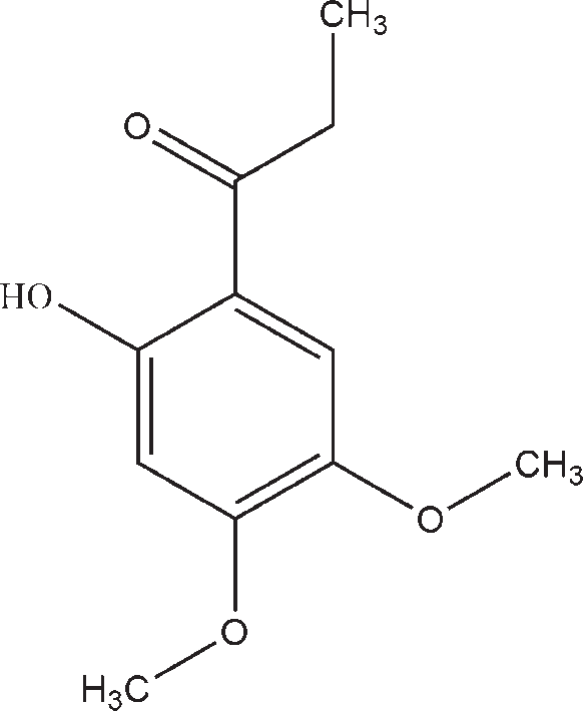


## Experimental

### 

#### Crystal data

C_11_H_14_O_4_
*M* *_r_* = 210.22
Monoclinic, 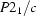

*a* = 7.1933 (7) Å
*b* = 9.4874 (12) Å
*c* = 17.198 (2) Å
β = 113.164 (5)°
*V* = 1079.1 (2) Å^3^
*Z* = 4
Mo *K*α radiation
μ = 0.10 mm^−1^
*T* = 293 K
0.31 × 0.16 × 0.14 mm

#### Data collection

Bruker SMART CCD diffractometer
Absorption correction: multi-scan (*SADABS*; Bruker, 1997 ▶) *T*_min_ = 0.066, *T*_max_ = 0.185
7549 measured reflections
2673 independent reflections
1749 reflections with *I* > 2σ(*I*)
*R*_int_ = 0.044

#### Refinement

*R*[*F* ^2^ > 2σ(*F* ^2^)] = 0.094
*wR*(*F* ^2^) = 0.295
*S* = 1.12
2673 reflections
136 parameters
H-atom parameters constrained
Δρ_max_ = 0.41 e Å^−3^
Δρ_min_ = −0.28 e Å^−3^

### 

Data collection: *SMART* (Bruker, 1997 ▶); cell refinement: *SAINT* (Bruker, 1997 ▶); data reduction: *SAINT*; program(s) used to solve structure: *SHELXS97* (Sheldrick, 2008 ▶); program(s) used to refine structure: *SHELXL97* (Sheldrick, 2008 ▶); molecular graphics: *SHELXTL* (Sheldrick, 2008 ▶); software used to prepare material for publication: *SHELXTL*.

## Supplementary Material

Crystal structure: contains datablocks global, I. DOI: 10.1107/S1600536809041798/hb5121sup1.cif

Structure factors: contains datablocks I. DOI: 10.1107/S1600536809041798/hb5121Isup2.hkl

Additional supplementary materials:  crystallographic information; 3D view; checkCIF report

## Figures and Tables

**Table 1:** Hydrogen-bond geometry (Å, °)

*D*—H⋯*A*	*D*—H	H⋯*A*	*D*⋯*A*	*D*—H⋯*A*
O1—H1⋯O2	0.82	1.86	2.577 (4)	146

**Fig. 1.**
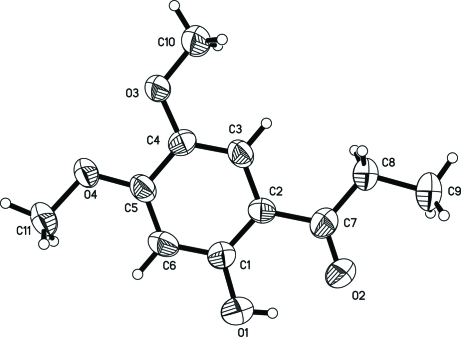
View of (I) with displacement ellipsoids drawn at the 30% probability level.

**Fig. 2.**
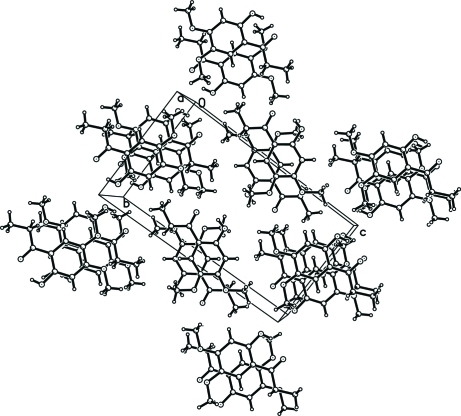
A view of the molecular packing for (I).

## supplementary crystallographic information

### Comment

Secondary metabolites in the plants of Trigonostemon xyphophylloides are mainly daphnane diterpenoids, phenanthrenones, alkaloids and coumarins (Tempeam *et al.*, 2005; Chen *et al.*, 2009). The plants in this family were used in folk medicine such as an emetic for food poisoning, a laxative and an anti-asthmatic, has also been used in the treatment of bloody and mucous sputum or stool. It was applied to reduce abscesses and to alleviate sprains, swelling and bruizes, is particularly effective in treating snake bites especially against snake neurotoxins. (Chuakul *et al.*, 1997; Tempeam *et al.*, 2002). The title compound was isolated from the 75% EtOH extract of the stems of Trigonostemon xyphophylloides which were collected from Jianfengling County, Hainan Province, P. *R*. China. We have undertaken the X-ray crystal structure analysis of the title compound in order to establish its molecular structure and relative stereochemistry.

The hydrogen bonds and angles are listed in Table 1.

### Experimental

Air-dried stems of Trigonostemon xyphophylloides (5.9 kg) were ground and percolated (3 × 2.5 h) with 75% EtOH at 333 K, which was suspended in 1.5 l water and then partitioned with petroleum ether, chloroform, ethyl acetate and n-BuOH, successively, yielding a petroleum ether extract, a chloroform extract, an ethyl acetate extract and a n-BuOH extract, respectively. The petroleum ether extract was subjected to a silica gel CC column using petroleum ether as first eluent and then increasing the polarity with EtOAc, to afford 20 fractions (A—T). Fraction D was further separated by column chromatography with a gradient of petroleum ether-EtOAc to give the title compound. The crude product was dissolved in small amount of ethyl acetate to obtain colourless blocks of (I) by slow evaporation of ethyl acetate solution at 298 K.

### Refinement

H atoms bonded to C atoms were palced in geometrically calculated position and were refined using a riding model, with *U*_iso_(H) = 1.2 *U*_eq_(C). H atoms attached to O atoms were found in a difference Fourier synthesis and were refined using a riding model, with the O—H distances fixed as initially found and with *U*_iso_(H) values set at 1.5 *U*eq(O).

### Crystal data

**Table:**

C_11_H_14_O_4_	*F*(000) = 448
*M**_r_* = 210.22	*D*_x_ = 1.294 Mg m^−^^3^
Monoclinic, *P*2_1_/*c*	Melting point: not measured K
Hall symbol: -P 2ybc	Mo *K*α radiation, λ = 0.71073 Å
*a* = 7.1933 (7) Å	Cell parameters from 2673 reflections
*b* = 9.4874 (12) Å	θ = 2.5–28.4°
*c* = 17.198 (2) Å	µ = 0.10 mm^−^^1^
β = 113.164 (5)°	*T* = 293 K
*V* = 1079.1 (2) Å^3^	Block, colourless
*Z* = 4	0.31 × 0.16 × 0.14 mm

### Data collection

**Table:**

Bruker SMART CCD diffractometer	2673 independent reflections
Radiation source: fine-focus sealed tube	1749 reflections with *I* > 2σ(*I*)
graphite	*R*_int_ = 0.044
ω scans	θ_max_ = 28.4°, θ_min_ = 2.5°
Absorption correction: multi-scan (*SADABS*; Bruker, 1997)	*h* = −9→9
*T*_min_ = 0.066, *T*_max_ = 0.185	*k* = −12→12
7549 measured reflections	*l* = −22→13

### Refinement

**Table:**

Refinement on *F*^2^	Primary atom site location: structure-invariant direct methods
Least-squares matrix: full	Secondary atom site location: difference Fourier map
*R*[*F*^2^ > 2σ(*F*^2^)] = 0.094	Hydrogen site location: inferred from neighbouring sites
*wR*(*F*^2^) = 0.295	H-atom parameters constrained
*S* = 1.12	*w* = 1/[σ^2^(*F*_o_^2^) + (0.1225*P*)^2^ + 1.1131*P*] where *P* = (*F*_o_^2^ + 2*F*_c_^2^)/3
2673 reflections	(Δ/σ)_max_ < 0.001
136 parameters	Δρ_max_ = 0.41 e Å^−^^3^
0 restraints	Δρ_min_ = −0.28 e Å^−^^3^

### Special details

**Table:**

Geometry. All e.s.d.'s (except the e.s.d. in the dihedral angle between two l.s. planes) are estimated using the full covariance matrix. The cell e.s.d.'s are taken into account individually in the estimation of e.s.d.'s in distances, angles and torsion angles; correlations between e.s.d.'s in cell parameters are only used when they are defined by crystal symmetry. An approximate (isotropic) treatment of cell e.s.d.'s is used for estimating e.s.d.'s involving l.s. planes.
Refinement. Refinement of *F*^2^ against ALL reflections. The weighted *R*-factor *wR* and goodness of fit *S* are based on *F*^2^, conventional *R*-factors *R* are based on *F*, with *F* set to zero for negative *F*^2^. The threshold expression of *F*^2^ > σ(*F*^2^) is used only for calculating *R*-factors(gt) *etc*. and is not relevant to the choice of reflections for refinement. *R*-factors based on *F*^2^ are statistically about twice as large as those based on *F*, and *R*- factors based on ALL data will be even larger.

### Fractional atomic coordinates and isotropic or equivalent isotropic displacement parameters (Å^2^)

**Table:**

	*x*	*y*	*z*	*U*_iso_*/*U*_eq_	
O1	0.3767 (5)	0.3553 (3)	0.52714 (19)	0.0723 (9)	
H1	0.3323	0.3757	0.4768	0.080*	
O2	0.1907 (5)	0.3117 (3)	0.36706 (18)	0.0763 (9)	
O3	0.2687 (4)	−0.2066 (3)	0.58386 (15)	0.0600 (8)	
O4	0.4475 (4)	−0.0386 (3)	0.71051 (14)	0.0581 (7)	
C1	0.3465 (5)	0.2161 (4)	0.5358 (2)	0.0497 (8)	
C2	0.2485 (5)	0.1275 (3)	0.4653 (2)	0.0435 (7)	
C3	0.2245 (5)	−0.0169 (4)	0.48170 (19)	0.0437 (7)	
H3	0.1624	−0.0776	0.4364	0.080*	
C4	0.2907 (5)	−0.0694 (3)	0.5629 (2)	0.0432 (7)	
C5	0.3876 (5)	0.0225 (4)	0.63263 (19)	0.0441 (7)	
C6	0.4134 (5)	0.1631 (4)	0.6178 (2)	0.0499 (8)	
H6	0.4764	0.2232	0.6633	0.080*	
C7	0.1702 (5)	0.1852 (4)	0.3788 (2)	0.0509 (8)	
C8	0.0636 (6)	0.0885 (4)	0.3045 (2)	0.0569 (9)	
H8A	−0.0529	0.0476	0.3111	0.080*	
H8B	0.1545	0.0120	0.3061	0.080*	
C9	−0.0065 (8)	0.1589 (5)	0.2186 (3)	0.0801 (14)	
H9A	−0.0699	0.0902	0.1752	0.080*	
H9B	0.1077	0.1990	0.2110	0.080*	
H9C	−0.1017	0.2319	0.2152	0.080*	
C10	0.1800 (6)	−0.3028 (4)	0.5156 (2)	0.0600 (10)	
H10A	0.1714	−0.3945	0.5376	0.080*	
H10B	0.2621	−0.3079	0.4831	0.080*	
H10C	0.0469	−0.2709	0.4801	0.080*	
C11	0.5335 (7)	0.0537 (5)	0.7830 (2)	0.0699 (12)	
H11A	0.5684	−0.0003	0.8340	0.080*	
H11B	0.4364	0.1246	0.7809	0.080*	
H11C	0.6527	0.0980	0.7821	0.080*	

### Atomic displacement parameters (Å^2^)

**Table:**

	*U*^11^	*U*^22^	*U*^33^	*U*^12^	*U*^13^	*U*^23^
O1	0.107 (2)	0.0456 (15)	0.0605 (17)	−0.0084 (14)	0.0294 (16)	−0.0016 (13)
O2	0.115 (3)	0.0534 (17)	0.0561 (16)	−0.0070 (16)	0.0287 (17)	0.0092 (13)
O3	0.0858 (18)	0.0402 (13)	0.0372 (12)	−0.0007 (12)	0.0062 (12)	0.0020 (10)
O4	0.0790 (17)	0.0490 (14)	0.0347 (12)	−0.0005 (12)	0.0098 (11)	−0.0045 (10)
C1	0.0556 (19)	0.0434 (18)	0.0509 (18)	0.0009 (15)	0.0219 (15)	−0.0022 (14)
C2	0.0482 (17)	0.0395 (16)	0.0422 (16)	0.0051 (13)	0.0170 (13)	−0.0005 (13)
C3	0.0472 (17)	0.0426 (17)	0.0377 (15)	0.0044 (13)	0.0129 (13)	−0.0046 (13)
C4	0.0444 (16)	0.0399 (16)	0.0413 (16)	0.0072 (13)	0.0125 (13)	−0.0007 (13)
C5	0.0459 (16)	0.0452 (17)	0.0371 (15)	0.0061 (14)	0.0120 (12)	−0.0036 (13)
C6	0.0551 (19)	0.0473 (19)	0.0437 (17)	0.0005 (15)	0.0155 (15)	−0.0091 (14)
C7	0.058 (2)	0.048 (2)	0.0461 (18)	0.0053 (15)	0.0200 (15)	0.0048 (15)
C8	0.069 (2)	0.057 (2)	0.0405 (17)	0.0013 (17)	0.0162 (16)	0.0066 (15)
C9	0.107 (4)	0.073 (3)	0.045 (2)	0.001 (3)	0.013 (2)	0.012 (2)
C10	0.078 (3)	0.0443 (19)	0.0439 (18)	0.0030 (17)	0.0091 (17)	−0.0015 (15)
C11	0.098 (3)	0.065 (3)	0.0358 (17)	−0.002 (2)	0.0149 (19)	−0.0100 (17)

### Geometric parameters (Å, °)

**Table:**

O1—C1	1.355 (4)	C6—H6	0.9300
O1—H1	0.8200	C7—C8	1.513 (5)
O2—C7	1.235 (4)	C8—C9	1.516 (5)
O3—C4	1.376 (4)	C8—H8A	0.9700
O3—C10	1.424 (4)	C8—H8B	0.9700
O4—C5	1.364 (4)	C9—H9A	0.9600
O4—C11	1.448 (4)	C9—H9B	0.9600
C1—C6	1.393 (5)	C9—H9C	0.9600
C1—C2	1.415 (5)	C10—H10A	0.9600
C2—C3	1.423 (5)	C10—H10B	0.9600
C2—C7	1.473 (5)	C10—H10C	0.9600
C3—C4	1.379 (4)	C11—H11A	0.9600
C3—H3	0.9300	C11—H11B	0.9600
C4—C5	1.423 (4)	C11—H11C	0.9600
C5—C6	1.385 (5)		
			
C1—O1—H1	109.5	C7—C8—C9	114.8 (3)
C4—O3—C10	116.8 (3)	C7—C8—H8A	108.6
C5—O4—C11	116.8 (3)	C9—C8—H8A	108.6
O1—C1—C6	117.2 (3)	C7—C8—H8B	108.6
O1—C1—C2	122.2 (3)	C9—C8—H8B	108.6
C6—C1—C2	120.7 (3)	H8A—C8—H8B	107.5
C1—C2—C3	117.4 (3)	C8—C9—H9A	109.5
C1—C2—C7	120.5 (3)	C8—C9—H9B	109.5
C3—C2—C7	122.0 (3)	H9A—C9—H9B	109.5
C4—C3—C2	121.9 (3)	C8—C9—H9C	109.5
C4—C3—H3	119.1	H9A—C9—H9C	109.5
C2—C3—H3	119.1	H9B—C9—H9C	109.5
O3—C4—C3	125.3 (3)	O3—C10—H10A	109.5
O3—C4—C5	115.3 (3)	O3—C10—H10B	109.5
C3—C4—C5	119.4 (3)	H10A—C10—H10B	109.5
O4—C5—C6	125.2 (3)	O3—C10—H10C	109.5
O4—C5—C4	115.3 (3)	H10A—C10—H10C	109.5
C6—C5—C4	119.4 (3)	H10B—C10—H10C	109.5
C5—C6—C1	121.1 (3)	O4—C11—H11A	109.5
C5—C6—H6	119.4	O4—C11—H11B	109.5
C1—C6—H6	119.4	H11A—C11—H11B	109.5
O2—C7—C2	120.2 (3)	O4—C11—H11C	109.5
O2—C7—C8	120.3 (3)	H11A—C11—H11C	109.5
C2—C7—C8	119.5 (3)	H11B—C11—H11C	109.5

### Hydrogen-bond geometry (Å, °)

**Table:**

*D*—H···*A*	*D*—H	H···*A*	*D*···*A*	*D*—H···*A*
O1—H1···O2	0.82	1.86	2.577 (4)	146
